# Clinical Holistic Medicine (Mindful,Short-Term Psychodynamic Psychotherapy Complemented with Bodywork) in the Treatment of Experienced Mental Illness

**DOI:** 10.1100/tsw.2007.67

**Published:** 2007-03-02

**Authors:** Søren Ventegodt, Suzette Thegler, Tove Andreasen, Flemming Struve, Lars Enevoldsen, Laila Bassaine, Margrethe Torp, Joav Merrick

**Affiliations:** ^1^ Quality of Life Research Center, Teglgårdstræde 4-8, DK-1452 Copenhagen K, Denmark; ^2^ Research Clinic for Holistic Medicine, Copenhagen, Denmark; ^3^ Nordic School of Holistic Medicine, Copenhagen, Denmark; ^4^ Scandinavian Foundation for Holistic Medicine, Sandvika, Norway; ^5^ Interuniversity College, Graz, Austria; ^6^ Zusman Child Development Center, Soroka University Medical Center, Ben Gurion University of the Negev, Beer-Sheva, Israel; ^7^ National Institute of Child Health and Human Development, Jerusalem, Israel; ^8^ Office of the Medical Director, Division for Mental Retardation, Ministry of Social Affairs, Jerusalem, Israel

**Keywords:** human development, complementary and alternative medicine, CAM, mental health, Denmark, short-term psychodynamic psychotherapy (STPP), holistic medicine, existential healing, bodywork, salutogenesis, Antonovsky

## Abstract

Short-term psychodynamic psychotherapy (STPP) complemented with bodywork improved 31 of 54 patients (57.4%, 95% CI: 43.21–70.77%) who rated themselves mentally ill before treatment. Calculated from this we find 1.41 < NNT < 2.31; we estimate NNH > 500. Of the 54 patients, 40% had already had traditional treatment that did not help them. Bodywork helped the patients to confront repressed painful feelings from childhood and this seemingly accelerated and improved the therapy. The patients received in average 20 sessions over 14 months at a cost of 1600 EURO. For the treatment responders, all measured aspects of life (on a five point Likert Scale) improved significantly, simultaneously, and radically: somatic health (from 2.9 to 2.3), self-esteem/relationship to self (from 3.5 to 2.3), relationship to partner (from 4.7 to 2.9 [no partner was rated as “6”]), relationship to friends (from 2.5 to 2.0), ability to love (from 3.8 to 2.4), self-assessed sexual ability (from 3.5 to 2.4), self-assessed social ability (from 3.2 to 2.1), self-assessed working ability (from 3.3 to 2.4), and self-assessed quality of life (from 4.0 to 2.3. Quality of life as measured with QOL5 improved (from 3.6 to 2.3 on a scale from 1 to 5; p < 0.001). This general improvement strongly indicated that the patient had healed existentially, i.e., had experienced what Aaron Antonovsky (1923–1994) called “salutogenesis”, defined as the process exactly the opposite of pathogenesis. For the treatment responders, the treatment provided lasting benefits, without the negative side effects of drugs. A lasting, positive effect might also prevent many different types of problems in the future.

## INTRODUCTION

Psychodynamic short-term therapy was proven successful in several meta-studies with mental health problems[1,2,3,4]. The therapy is believed to work even when the patient is suffering from schizophrenia[5]. Since 1997, in our clinical work, we have made psychodynamic therapy more efficient by including bodywork[6] and, in this way, have accelerated the therapeutic process by helping the patient to confront repressed feelings from childhood trauma.

## METHODS

In 2004–2005, we treated 54 patients (15–60 years of age) who rated themselves as mentally ill on a 5-point Likert scale of self-assessed mental health when they came to our clinic. We measured the outcome with a validated questionnaire (QOL5)[7] and used the bodywork of Marion Rosen[8]. We included the patients (15–60 years, both genders) that spontaneously appeared in our clinic, which is open towards the street. Of these patients, 40% had already had traditional psychiatric treatment for a mental disease (mostly depression and schizophrenia) without feeling sufficiently helped. They rated themselves on assessment an average of 3.7 (standard deviation 0.76) on the 5-point Likert scale of self-assessed mental health. The patients received, on average, 20 sessions over 14 months and paid 1,600 EURO.

## RESULTS

We found that almost all patients had spontaneous regression to early childhood trauma when bodywork[8] was used in combination with psychodynamic therapy. Bodywork helped the patient to confront the repressed, often very unpleasant, feelings from childhood trauma(s), which accelerated therapy. Of the 54 patients, 31 (57.4%, 95% CI: 43.21–70.77%) who rated themselves mentally ill before treatment did not do so after treatment (estimated: $1.41 < NNT < 2.31$)(NNT = Number Needed to Treat); i.e., 54 patients felt mentally ill before treatment (rating 4 or 5 on the 5-point Likert scale of self-assessed mental health of QOL5) and 31 patients did not feel mentally ill after treatment (rating 1, 2, or 3 on the Likert scale). Six patients still felt mentally ill after treatment (rating 4 or 5); 17 were nonresponders on follow-up 1 year after.

Most interestingly, for the treatment responders, all measured aspects of life improved significantly, simultaneously, and radically (see Table 1): somatic health (from 2.9 to 2.3), self-esteem/relationship to self (from 3.5 to 2.3), relationship to partner (from 4.7 to 2.9 [no partner was rated as “6”]), relationship to friends (from 2.5 to 2.0), ability to love (from 3.8 to 2.4), self-assessed sexual ability (from 3.5 to 2.4), self-assessed social ability (from 3.2 to 2.1), self-assessed working ability (from 3.3 to 2.4), and self-assessed quality of life (from 4.0 to 2.3) (see Table 1). Quality of life as measured with QOL5 improved (from 3.6 to 2.3 on a scale from 1 to 5; $p < 0.001$). This general improvement strongly indicated that the patient had healed existentially, i.e., had experienced what Aaron Antonovsky (1923–1994) called “salutogenesis”[9], defined as the process exactly the opposite of pathogenesis.

**TABLE 1**
**Patients who Changed from Feeling Mentally Ill to Mentally Well**

| | Paired Differences* | | | | | t | df | Significance (Two–Tailed) |
|---|---|---|---|---|---|---|---|---|
| | Mean | Std. Deviation | Std. Error Mean | 95% CI of Difference | | | | |
| | | | | Lower | Upper | | | |
| Physical health | 0.6000 | 0.89443 | 0.16330 | 0.2660 | 0.9340 | 3.674 | 29 | 0.001 |
| Mental health | 1.9677 | 0.79515 | 0.79515 | 1.6761 | 2.2594 | 13.778 | 30 | 0.000 |
| Self-esteem | 1.2258 | 1.11683 | 1.11683 | 0.8161 | 1.6355 | 6.111 | 30 | 0.000 |
| Relation to friends | 0.5161 | 0.92632 | 0.92632 | 0.1764 | 0.8559 | 3.102 | 30 | 0.004 |
| Relation to partner | 1.8065 | 2.27185 | 2.27185 | 0.9731 | 2.6398 | 4.427 | 30 | 0.000 |
| Ability to love | 1.3548 | 1.60309 | 1.60309 | 0.7668 | 1.9429 | 4.706 | 30 | 0.000 |
| Sexual ability | 1.0323 | 1.35361 | 1.35361 | 0.5358 | 1.5288 | 4.246 | 30 | 0.000 |
| Social ability | 1.1613 | 1.12833 | 1.12833 | 0.7474 | 1.5752 | 5.730 | 30 | 0.000 |
| Work ability | 0.9000 | 1.06188 | 1.06188 | 0.5035 | 1.2965 | 4.642 | 29 | 0.000 |
| Quality of life | 1.7097 | 1.03902 | 1.03902 | 1.3286 | 2.0908 | 9.162 | 30 | 0.000 |

*Paired samples T-test.

The therapy was found to be safe, as none of more than 500 patients in our clinic over the past years have complained of any serious side effects (standard questionnaires) and, as none was harmed, we estimated the Number Needed to Harm (NNH) > 500.

The data were collected immediately after treatment and 1 year after. The reported data is from the last measurement we have of the patient. The second follow-ups showed that the mentally ill patients gained from the therapy and also retained the feeling of help 1 year later (for the responders).

## DISCUSSION

An important part of this existential healing happened in a few, very intense days of feeling very bad, when painful and repressed material from old childhood trauma(s) reappeared in the consciousness of the patient. These existential crises were not dangerous, but the patient needed intensive support (sometimes 24 h/day) during this period. It can be an ethical question whether or not putting patients into this emotional pain is acceptable, but most patients are very grateful when they felt cured afterwards.

We do believe that bodywork, and the recent development in psychodynamic therapy making it more “holistic”[6], has made it more “mindful”, less painful, and much more enjoyable. A strong philosophical focus on the meaning of life of the patient, his/her talents, and positive abilities has contributed to this.

Touching the patient is not allowed in many psychotherapeutic communities and, as the therapeutic touch seems to be the magical key to accelerate healing and Antonovsky-salutogenesis[9], these restricted rules might not be in the best interest of the patient and should therefore be reconsidered. Body therapy is often done with the patient naked or almost naked, and this might be considered as an ethical problem in many countries.

Another problem is that we need to make the study controlled, with randomization to standard treatment, and objective diagnosis. We also need to include more patients in the study and specific groups of mentally ill patients.

## CONCLUSIONS

The treatment of mental health patients with “clinical holistic medicine” (psychodynamic short-term therapy complemented with bodywork) seems to be extremely efficient in helping the mentally ill patient to improve by inducing existential healing, the so-called Antonovsky salutogenesis[9]. After only 20 sessions of therapy, the quality of life had improved dramatically, the self-esteem had improved, the relation to partner and friends had improved, and even self-assessed somatic health and self-assessed working ability had improved. It has no observed side effects and its results were permanent, judging from a 1-year follow-up.

We estimated the NNT of “clinical holistic medicine” with mentally ill patients to be 1.41–2.31 ($1.41 < NNT < 2.31$) and the $NNH > 500$.

## ACKNOWLEDGMENTS

This study was supported by grants from IMK Almene Fond. The quality of life research was originally approved by the Copenhagen Scientific Ethical Committee under number (KF)V.100.2123/91 and later correspondence.
